# Calcification of the Sphenomandibular Ligament: A Rare Case of Restricted Mouth Opening in a Pediatric Patient

**DOI:** 10.5334/jbsr.3749

**Published:** 2025-02-18

**Authors:** Sarah Kechtban, Pierre-Antoine Poncelet, Timothée Dontaine

**Affiliations:** 1 Department of Maxillo‑Facial Surgery and Facial Reconstruction, Grand Hôpital de Charleroi, Belgium; 2 Department of Radiology, Grand Hôpital de Charleroi, Belgium

**Keywords:** Sphenomandibular, ligament, calcification, ossification, trismus, limited mouth opening, pediatric

## Abstract

This report aims to present a very rare case of progressive limited mouth opening (trismus) in an 8‑year‑old child caused by calcification of the sphenomandibular ligament. This case highlights the difficulty of diagnostic and therapeutic challenges associated with this disorder. We discuss the detailed clinical and radiological approach, including the imaging techniques used to identify the calcification, as well as the treatment option chosen, namely maxillofacial physiotherapy. This report provides new information to the existing literature on this rare entity and to provide useful information for the clinical management of similar cases.

*Teaching point:* Sphenomandibular ligament ossification is a very rare cause of limitation of mouth opening and can be a challenging diagnosis.

## Case History

An 8‑year‑old female was referred to the maxillofacial surgery department for evaluation of a rapidly progressive mouth‑opening limitation. The patient did not present with history of pain or systemic complaints. There was no medical nor surgical history, or major maxillofacial trauma. She reported pain during specific masticatory movements. A physical examination revealed a limited mouth opening of 17 mm.

**Figure 1:**
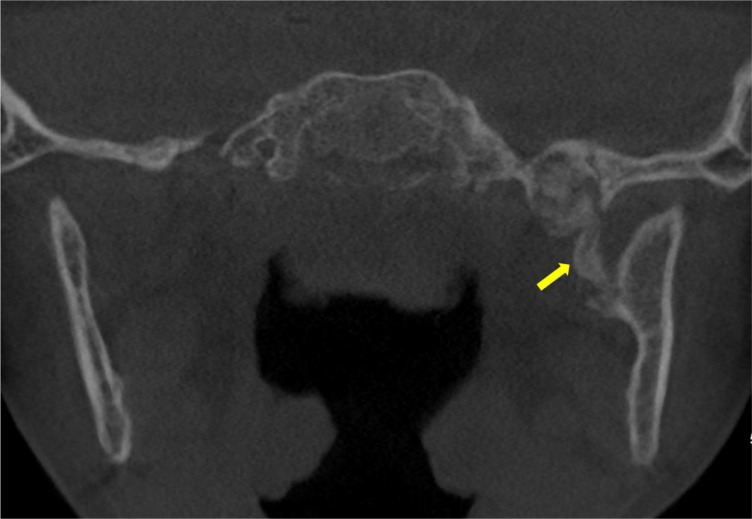
Unenhanced CT scan showing an ossified structure located between the left mandibular ramus and the spine the left sphenoid (arrow).

A computed tomography (CT) of the face revealed an oblique bony hypertrophy in the medial aspect of the left mandibular ramus (Figure 1). This multi‑fragmentary calcification connected the spine of the sphenoid bone and the Spix spine. It appeared to have a mass effect on the origin of the foramen ovale, with potential compression of the V3 nerve.

**Figure 2:**
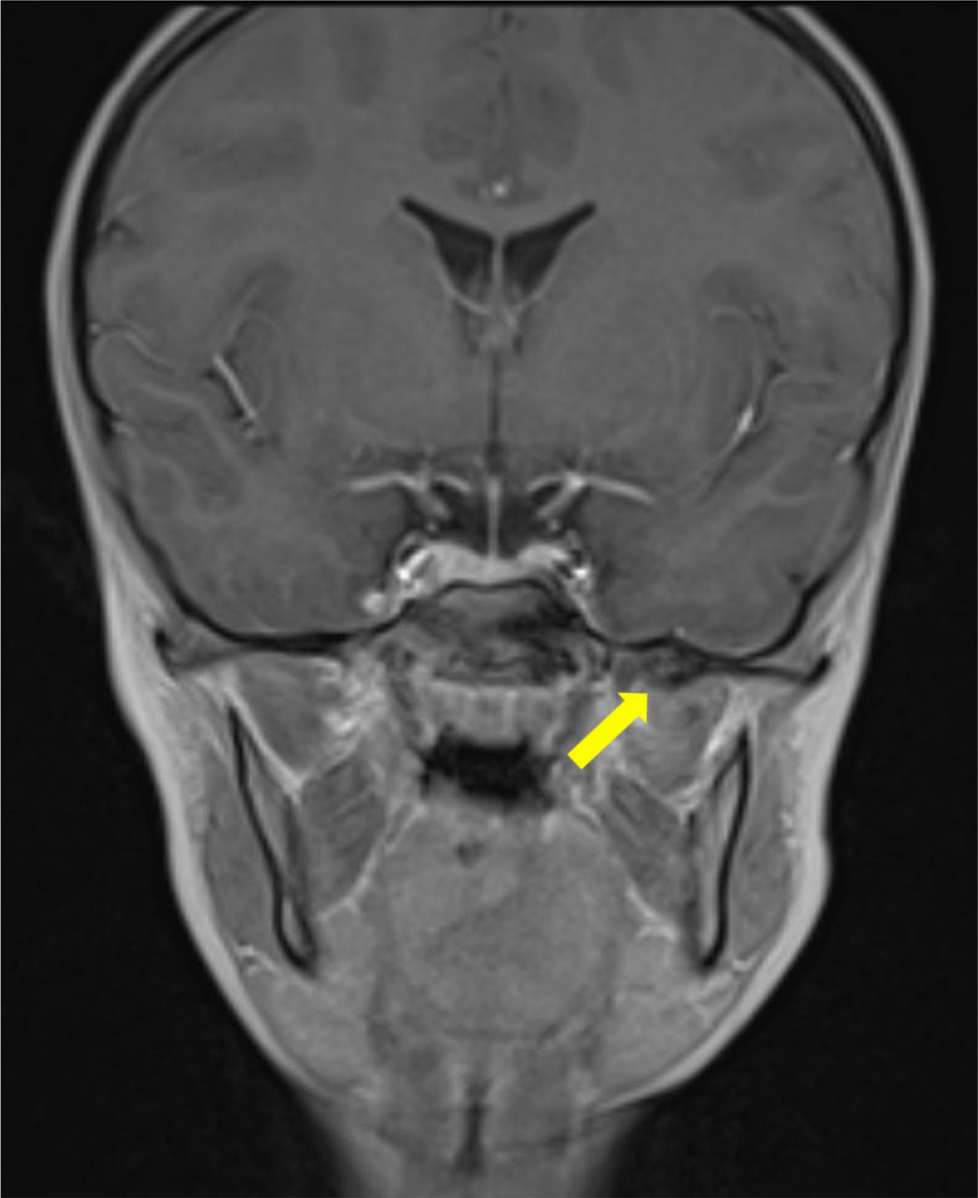
Contrast enhanced coronal T1 Dixon MRI scan showing the bony signal of the bony structure of the sphenoid spine (arrow) with no enhancement on the fat‑suppressed reconstructions (not shown).

**Figure 3:**
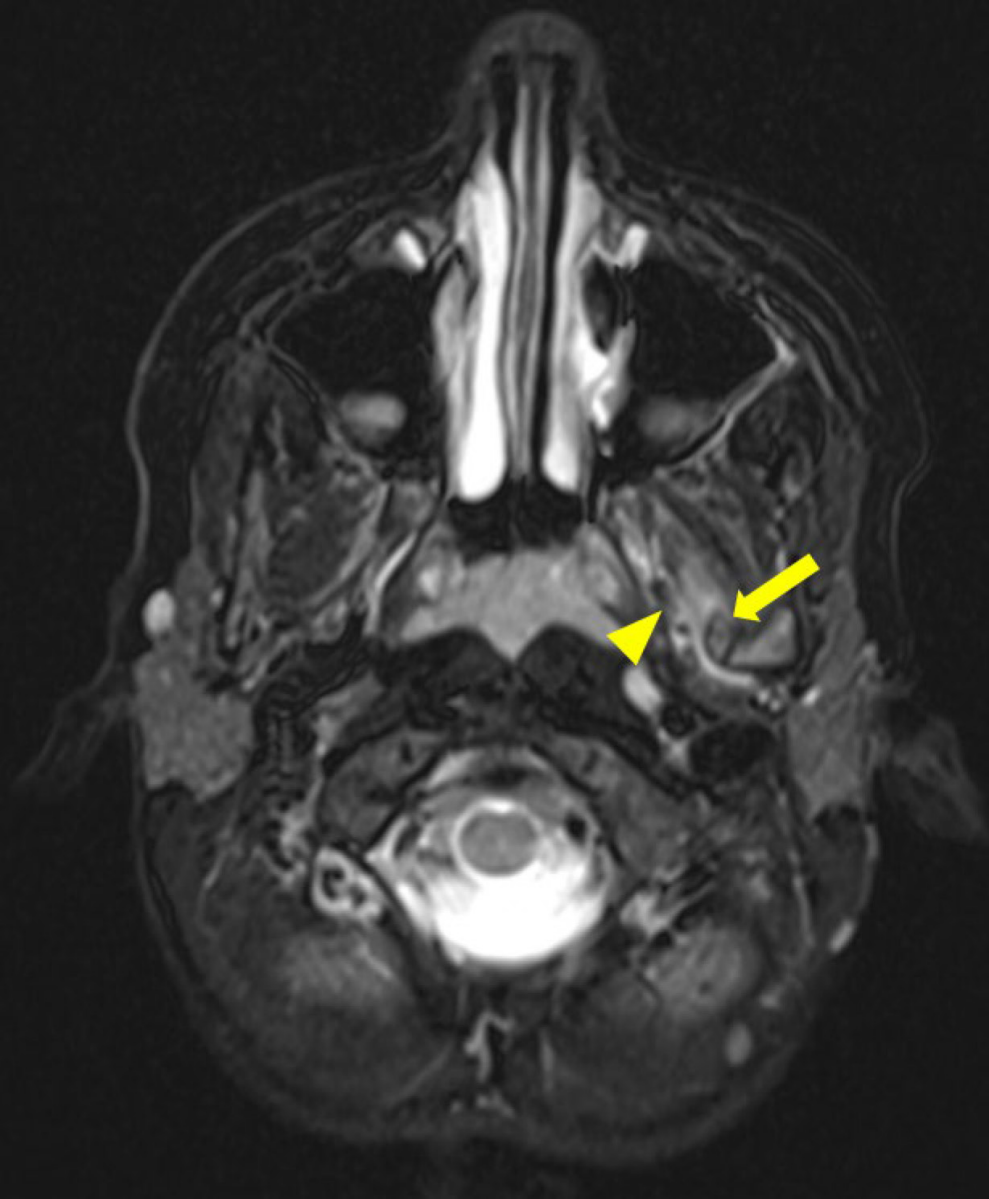
Axial T2 Dixon MRI scan showing edema infiltrating the lateral pterygoid muscles fibers (arrowhead) close to the ossified sphenomandibular ligament (arrow).

Magnetic resonance imaging (MRI) demonstrated a bony signal intensity of this ossified structure located between the left mandibular ramus and the spine of the left sphenoid (Figure 2). There was a reactive edema around the periphery of the structure, partially infiltrating the fibers of the lateral pterygoid muscle (Figure 3). There was an absence of a cartilaginous cap, suggesting an exostosis.

The path of ossification and the specific anatomical features suggested calcification of the sphenomandibular ligament (SML).

## Comments

The SML is one of the three extrinsic ligaments of the temporomandibular joint. It is a band of inextensible fibrous connective tissue that connects the spine of the sphenoid bone to the spine of the mandible. The SML has a role in limiting the range of mouth opening movements and supporting the joint during mastication. The SML is in close contact with the inferior alveolar nerve and the mylohyoid nerve.

Calcification of the SML is an extremely rare cause of trismus, with only two documented cases in the literature: one in a 7‑year‑old and the other in a 46‑year‑old patient.

Initial management of SML calcification constitutes maxillofacial physiotherapy. However, this non‑surgical approach proved ineffective in both cases mentioned above. The authors therefore proposed surgical resection of the band, with immediately improved mouth opening.

Following the parents’ request for the least invasive treatment, maxillofacial physiotherapy was used in the present case, resulting in mouth opening improvement to 30 mm after 6 months.

The patient is currently undergoing quarterly follow‑ups. If mouth opening worsens again, a surgical resection of the ligament will be suggested [1].

This case highlights the importance of multidisciplinary assessment and an individualized therapeutic approach in the management of a rare cause of mouth opening restrictions in children.
